# The use of ^18^F-Fluoro-deoxy-glucose positron emission tomography (^18^F-FDG PET) as a non-invasive pharmacodynamic biomarker to determine the minimally pharmacologically active dose of AZD8835, a novel PI3Kα inhibitor

**DOI:** 10.1371/journal.pone.0183048

**Published:** 2017-08-14

**Authors:** Juliana Maynard, Sally-Ann Emmas, Francois-Xavier Ble, Herve Barjat, Emily Lawrie, Urs Hancox, Urszula M. Polanska, Alison Pritchard, Kevin Hudson

**Affiliations:** 1 Personalised Healthcare & Biomarkers, AstraZeneca, Cheshire, United Kingdom; 2 Drug Safety and Metabolism iMED, AstraZeneca, Cheshire, United Kingdom; 3 Oncology Imed, Astrazenenca, Cheshire, United Kingdom; Stanford University, UNITED STATES

## Abstract

**Background:**

The phosphatidyl inositol 3 kinase (PI3K), AKT and mammalian target of rapamycin (mTOR) signal transduction pathway is frequently de-regulated and activated in human cancer and is an important therapeutic target. AZD8835 is a PI3K inhibitor, with selectivity against PI3K α and δ isoforms, which is currently in Phase 1 clinical trials. ^18^F-Fluoro-deoxy-glucose positron emission tomography (^18^F-FDG PET) is a non-invasive pharmacodynamic imaging biomarker that has become an integral part of drug development. It has been used widely with PI3K inhibitors both clinically and pre-clinically because of the role of the PI3K pathway in glucose metabolism. In this study we investigated the potential of ^18^F-FDG PET as a non-invasive pharmacodynamic biomarker for AZD8835. We sought to understand if ^18^F-FDG PET could determine the minimally effective dose of AZD8835 and correlate with other pharmacodynamic biomarkers for validation of its use in clinical development. ^18^F-FDG PET scans were performed in nude mice in the BT474C breast xenograft model. Mice were fasted prior to imaging and static ^18^F-FDG PET was performed. Treatment groups received AZD8835 by oral gavage at a dose volume of 10ml/kg. Treatment groups received either 3, 6, 12.5, 25 or 50mg/kg AZD8835. Tumour growth was monitored throughout the study, and at the end of the imaging procedure, tumours were taken and a full pharmacodynamic analysis was performed.

**Results:**

Results showed that AZD8835 reduced ^18^F-FDG uptake at a dose of 12.5, 25 and 50mg/kg with no significant reduction at doses of 3 and 6mg/kg. These results were consistent with other pharmacodynamics biomarkers measured and show ^18^F-FDG PET as a sensitive biomarker with the ability to determine the minimal effective dose of AZD8835.

**Conclusions:**

Our pre-clinical studies support the use of ^18^F-FDG PET imaging as a sensitive and non- invasive pharmacodynamic biomarker (understanding the role of PI3K signalling in glucose uptake) for AZD8835 with a decrease in ^18^F-FDG uptake observed at only two hours post treatment. The decrease in ^18^F-FDG uptake was dose dependent and data showed excellent PK/PD correlation. This data supports and parallels observations obtained with this class of compounds in patients

## Introduction

The phosphatidyl inositol 3 kinase (PI3K), AKT and mammalian target of rapamycin (mTOR) signal transduction pathway is frequently de-regulated and activated in human cancer and is an important therapeutic target [1]. Activation can occur by a variety of mechanisms including mutations in PIK3CA, PIK3R1 and AKT, loss of PTEN, or amplification of receptor tyrosine kinases such as HER2 [2]. Mutations in PIK3CA are estimated to be present in approximately 40% of hormone receptor positive breast cancers [3].

No drugs aimed specifically at cancers with PIK3CA mutations have been approved by the FDA to date, although several PI3K agents targeting the PIK3CA gene product, targeting PI3Kα, have entered into Phase 1 clinical trials [4] including BYL719 (Novartis) and GDC-0032 (Genentech). BYL719 is an α-specific PI3K inhibitor and entered into clinical trials in 2010 [5] to assess the therapeutic potential for treating cancers in which the PIK3CA gene is mutated or amplified. GDC-0032 is also a selective inhibitor of PI3K with reduced inhibitory activity against PI3Kβ and preferentially inhibits PIK3CA mutant cells relative to cells with wild type PI3K. It has been in clinical trials since 2012 [6].

AZD8835 (AstraZeneca) is a further example of a PI3K inhibitor, with selectivity against PI3K α and δ isoforms, which is currently in Phase 1 clinical trials. It selectively inhibits wild type and mutant forms of PI3Kα with equivalent potency and induces apoptosis and growth inhibition in mutant PIK3CA tumour models [7,8].

A key element in the clinical success of agents, such as AZD8835, is the use of robust sensitive pharmacodynamic biomarkers giving accurate information on target engagement to provide confidence that the candidate drug exposure and pharmacological activity in the target organ is being achieved [9]. As well as target engagement, pharmacodynamic biomarkers can also provide important information in guiding the dose escalation process and determining the optimum biological dose of the candidate compound. To have a robust surrogate marker to define and select a biologically active dose is currently one of the key gaps in the development of PI3K/AKT/mTOR inhibitors [4].

The use of pharmacodynamic biomarkers are a critical tool not only in clinical trials but also in pre-clinical studies. In this setting methods which allow measurement of target interactions enable better understanding of the PK/PD relationship of therapeutic agents and provide information that is critical in understanding the biological effects.

^18^F-Fluoro-deoxy-glucose positron emission tomography (^18^F-FDG PET) is a non-invasive pharmacodynamic imaging biomarker that has become an integral part of drug development. It is broadly accepted as a translational biomarker for disease progression and therapeutic response [10, 11]. It has been used widely with PI3K inhibitors both clinically and pre-clinically because of the role of the PI3K pathway in glucose metabolism [12, 13].

Specifically it has also been used in clinical trials with both PI3Kα inhibitors BYL719 and GDC-0032 and shows convincing biomarker modulation in both clinical dose cohort studies [14].

Pre-clinical ^18^F-FDG PET studies are a means to validate and optimise PET imaging protocols for intended use in clinical trials and to give biological characterisation of the candidate compound [15]. Pre-clinical validation and optimisation of the ^18^F-FDG PET biomarker is encouraged prior to incorporation into clinical trials [16].

The aim of this study was to investigate the potential of ^18^F-FDG PET as a non-invasive pharmacodynamic biomarker for AZD8835. We sought to understand if ^18^F-FDG PET could determine the minimally effective dose of AZD8835 and correlate with other pharmacodynamic biomarkers for validation of its use in clinical development.

## Materials and methods

### Animal model

Female nude mice (nu/nu:Alpk) were bred at AstraZeneca and maintained in rooms under controlled conditions of temperature (19–23°C) and humidity (55% ± 10%), photoperiod (12h light/12h dark) and air exchange, with food and water provided *ad libitum*. The facilities have been approved by the Home Office and meet all current regulations and standards of the UK.

Mice underwent subcutaneous inoculation of the PI3Kαdriven BT474C breast carcinoma human tumour cell line (derived in AZ from BT474 (ATCC HTB-20)) tumours passaged in mice. Cells were implanted on the left flank in a volume of 0.1ml of RPMI medium containing 5.0 x 10^6^ cells.

Animals were supplemented with 0.36mg/60 day 17β estradiol pellets (Innovative Research of America) 1 day prior to cell implantation.

For *in-vivo* implant, cells were harvested following treatment with 0.05% trypsin (Invitrogen) in EDTA solution followed by a suspension in basic medium and three washes in phosphate-buffered saline (Invitrogen). Only single-cell suspensions of greater than 90% viability, as determined by trypan blue exclusion, were used for injection.

### Imaging studies

When mean tumour sizes reached approximately 0.2cm^3^, mice were randomised into control and treatment groups. Tumours were measured using bilateral vernier calliper measurements using the formula (length x width) x √(length x width) x (π/6).

Treatment groups received AZD8835 by oral gavage at a dose volume of 10ml/kg. Treatment groups received either 3, 6, 12.5, 25 or 50mg/kg AZD8835. Prior to the dose ranging study a single dose of 12.5mg/kg was used to assess if a reduction in ^18^F-FDG uptake existed prior to dose response progression. AZD8835 was prepared and solubilised in HPMC/Tween solution (0.5% methylcellulose, 0.1% polysorbate 80). Mice were dosed 2 hours prior to imaging. On the day of imaging, food was withdrawn at 7am so mice were fasted for at least four hours prior to ^18^F-FDG injection. Blood glucose concentration was measured before vehicle or AZD8835 administration and after PET scanning. Blood glucose concentrations were measured using an AccuChek metre (AVIVA, Roche). Anaesthesia was induced using isoflorane delivered in 100% oxygen (~1.5% isoflorane, 3L oxygen). Respiration and temperature were maintained throughout, with body temperature being maintained at 36–37°C.

### ^18^F-FDG PET imaging

^18^F-FDG was supplied by PETNET solutions, Nottingham, UK. Imaging was performed using the Inveon Multimodality ^™^ PET scanner from Siemens Medical Solutions.

Mice received approximately 15MBq ^18^F-FDG administered as an i.v. bolus. Following ^18^F- FDG injection, anaesthesia was maintained for a 45-minute uptake period followed by a 20-minute emission PET scan. Animals were humanely euthanised following scanning. Data were acquired using Inveon Acquisition Workplace (IAW) software (Siemens) version 1.4.3 and analysed using Inveon Reconstruction Workplace (IRW) software (Siemens) version 2.2.0. The performance of the scanner has been documented previously [17]. Images were reconstructed using the order subset expectation maximisation (OSEM)/maximum a posterior (MAP) algorithm. Regions of interest (ROI’s) were drawn manually using the 3D visualisation package of IRW software. Data were expressed as the maximum standardised uptake value (MaxSUV). MaxSUV was calculated using the formula described by Gambhir *et al*, where ID is the injected activity [18].

### Biodistribution analysis

Blood, muscle, lung, liver, tumour, bone and tail were collected for biodistribution analysis. Samples were weighed and weights were recorded. Tissue samples were counted in a gamma counter (Perkin Elmer, 1480, Wizard 3) for 20s per sample. The gamma counter provides a “counts per minute” parameter. These data were imported into an Excel spreadsheet where the counts per minute were converted into activity by conversions into disintegrations per minute; by multiplying the efficiency of the gamma counter for ^18^F. Activity was decay corrected to the time of injection and converted into a concentration using the *ex-vivo* tissue weight (in kilobecquerel per gram). Tissue weights were imported into the Excel spreadsheet. All mice in which ^18^F-FDG radioactivity in the tail exceeded 10% of the injected dose were excluded from analysis.

### Pharmacokinetic analysis

Blood was removed via cardiac puncture and placed in a plasma collection tube with lithium heparin. Blood was centrifuged at 12,000 rpm for 5 minutes, the plasma aspirated and stored at -20°C.

Plasma samples were extracted by protein precipitation in methanol. Following centrifugation, the supernatants were mixed with water in a ratio of 1 in 10 (v/v). Extracts were analysed by high performance liquid chromatography/ mass spectrometry using a reversed phase Gemini column (Phenomenex) and a gradient mobile phase column containing water/methanol/ formic acid. Peaks were detected using a MicroMass/Waters MS technology Ultima mass spectrometer.

### Pharmacodynamic analysis

Following removal tumours were cut in half. Half was placed in 4% buffered formalin for 24 hours before being stored in 70% ethanol. Tumours in ethanol were used for immunohistochemical analysis.

The remaining half of the tumour was snap frozen in liquid nitrogen and stored at -80°C. Frozen tumours were used for MesoScale Discovery and ELISA assay analysis.

#### Immunohistochemistry

For quantification of pPRAS40 expression via immunohistochemistry, specimens were embedded in paraffin and cut at a thickness of 3–5μM. Slides were incubated with Rabbit Mab anti-phospho PRAS40 (Thr 246) (CD77D7; dilution 1:200) for detecting pPRAS40 expression and detection of immunostaining was performed using a Dako Rabbit Envision HRP polymer kit (Dako, Cat# 51699).

#### Meso Scale Discovery Assay and ELISA

Quantification of AKT was performed by Meso Scale Discovery Assay (Phospho-AKT-Ser473 Ab: Mesoscale.com D20MN-3 & cleaved-caspase-3: Mesoscale.com K151CFD-1) and quantification of pPRAS40 by sandwich ELISA (Biosource # KH00421). A protein assay was performed prior to both assays (Pierce Protein Assay Reagent A (233228) and BCA Protein Assay Reagent B (233224).

### Statistical analysis

Data are reported as the mean ± SEM unless otherwise stated. Statistical analyses were performed using Graph Pad Prism (v4.02) and group means compared using a two-sided t-test or in dose response studies a monotonic dose response relationship was assumed and a William’s test for variance performed. All studies were performed according to good statistical practice and an in-house statistician appropriately powered all studies performed.

## Results

### ^18^F-FDG PET demonstrates target engagement 2 hours after dosing with AZD8835

**Fig 1:**
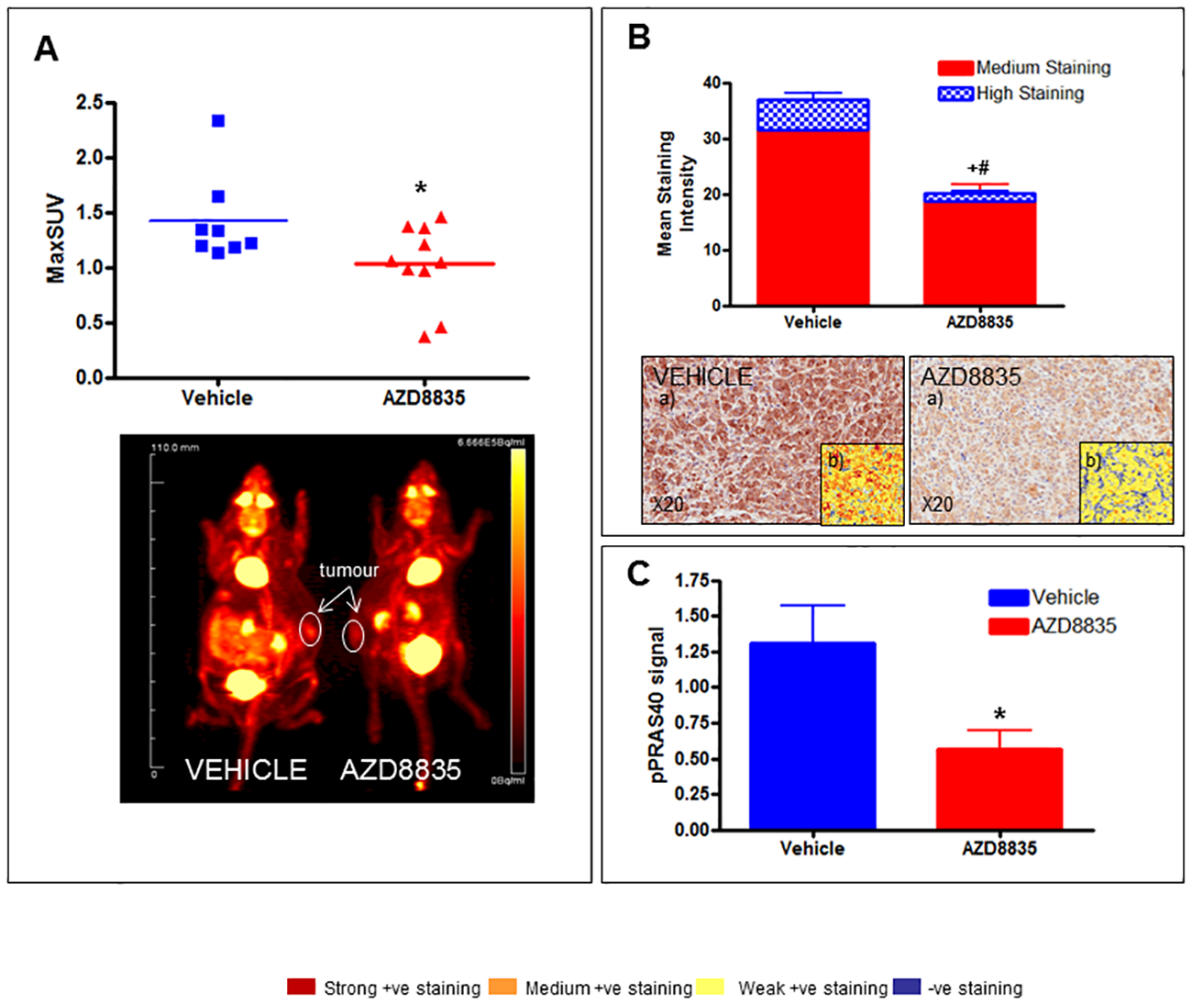
A single dose of AZD8835 (12.5mg/kg) reduces ^18^F-FDG uptake and pPRAS40 expression 2 hours after dosing in the BT474C model. A) tumour ^18^F-FDG uptake following AZD8835 dosing (individual animal data). *p<0.05. B) Immunohistochemistry pPRAS40 data following AZD8835 dosing (mean ± SEM) a) = representative image at x20 magnification; b) = overlay of image anaylsis:; +#p<0.05 vs vehicle. C) ELISA pPRAS40 data following AZD8835 dosing (mean± SEM). *p<0.05.

The potential of ^18^F-FDG PET to be a pharmacodynamic biomarker for AZD8835 was initially measured by its ability to reduce ^18^F-FDG uptake after a single dose of AZD8835 (12.5mg/kg). 12.5mg/ kg is a sub-optimal efficacious dose in efficacy studies [8]. After 2 hours of dosing there was a significant decrease in tumour ^18^F-FDG uptake seen with both imaging and biodistribution data (27% and 23% respectively) (Fig 1A and Table 1). MaxSUV from imaging data was 1.42 ± 0.12 and 1.03 ± 0.11 in the vehicle and AZD8835 treated group respectively. %ID/g in the tumour from biodistribution data was 2.39 ± 0.12 and 1.84 ± 0.19 in the vehicle and AZD8835 treated group respectively. There were no significant differences in any other tissues from biodistribution data (Table 1).

**Table 1:** 18F-FDG uptake in the BT474C xenograft model in background tissues following dosing with AZD8835 (12.5mg/kg) or vehicle. *p<0.05 vs vehicle.

Group	%ID/g				
	Tumour	Blood	Muscle	Lung	Liver
Vehicle (n = 8)	2.39 ± 0.12	0.85 ± 0.07	0.53 ± 0.04	3.80 ± 0.36	1.44 ± 0.09
AZD8835 12.5mg/kg (n = 10)	1.84 ± 0.19	0.96 ± 0.08	0.70 ± 0.11	2.99 ± 0.35	1.53 ± 0.15

Pharmacokinetic analysis quantified circulating AZD8835 in the blood (2.42μM ± 0.12). Pharmacodynamic analysis using immunohistochemistry (IHC) and ELISA analysis showed suppression of downstream signalling using phosphorylation of PRAS40 as a read-out of PI3K pathway activity. The IHC H score of formalin fixed tumours measuring pPRAS40 expression was significantly lower following administration of AZD8835 being 130 ± 7.72 and 99.01 ± 7.60 in the vehicle and AZD8835 treated group respectively (Fig 1B). ELISA measurements of pPRAS40 also showed a significant decrease in pPRAS40 signal following administration of AZD8835 and was 57% lower in the AZD8835 treated group compared to vehicle (Fig 1B). Blood glucose concentration was also measured pre-and post-treatment to ensure that there were no significant differences in systemic glucose following AZD8835 administration. A paired t-test showed that were no significant differences in blood glucose concentration post treatment in either the vehicle or the 12.5mg/kg AZD8835 treated group (p = 0.31 and 0.11 respectively), showing no competing and systemic blood glucose effects at this time.

### ^18^F-FDG PET can determine the minimally effective dose of AZD8835

**Fig 2:**
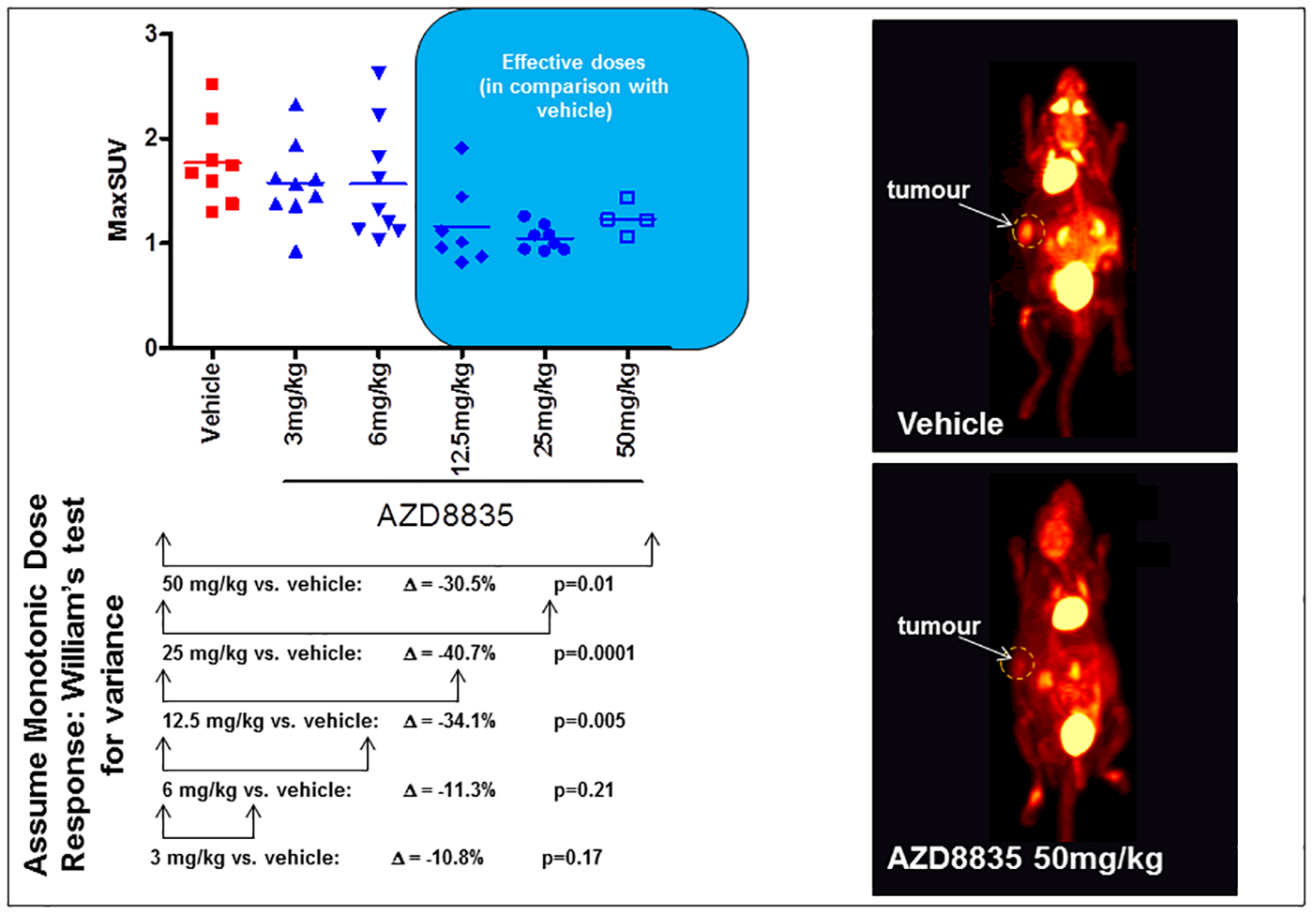
Pharmacodynamic knockdown of PI3K pathway activity using ^18^F-FDG PET is observed 2 hours after dosing in the BT474C model at 12.5, 25 and 50mg/kg. Tumour ^18^F-FDG uptake following AZD8835 dosing (individual animal data). (vehicle n = 8; AZD8835 3mg/kg n = 9; AZD8835 6mg/kg n = 9; AZD8835 12.5mg/kg n = 7; AZD8835 25mg/kg n = 8; AZD8835 50mg/kg n = 4). Representative images demonstrate the decrease in ^18^F-FDG uptake at the highest dose of 50mg/kg compared to vehicle.

Expanding on the initial single dose study, we then measured ^18^F-FDG uptake following a series of AZD8835 doses. After 2 hours of dosing there was a significant reduction in MaxSUV tumour ^18^F-FDG uptake following administration of AZD8835 at 12.5, 25 and 50mg/kg compared to vehicle (34.1%, 40.7% and 30.1% reduction in ^18^F-FDG uptake respectively compared to vehicle) (Fig 2). There was no significant reduction in ^18^F-FDG uptake at 3 and 6mg/kg AZD8835 compared to vehicle (11.3% and 10.8% reduction in ^18^F-FDG uptake respectively compared to vehicle). Biodistribution data also showed that there was a significant reduction in tumour ^18^F-FDG uptake at 12.5, 25 and 50mg/kg (Table 2). There was also a significant increase in ^18^F-FDG in the blood at 25 and 50mg/kg as well as in the muscle and liver at 50mg/kg (Table 2).

**Table 2:** 18F-FDG uptake in the BT474C xenograft model in background tissues following dosing with AZD8835 (3,6,12.5, 25 and 50mg/kg) or vehicle. *p<0.05 vs vehicle.

Group	%ID/g				
	Tumour	Blood	Muscle	Lung	Liver
Vehicle (n = 8)	3.72 ± 0.28	0.69 ± 0.05	0.78 ± 0.07	3.88 ± 0.58	1.61 ± 0.18
AZD8835 3mg/kg (n = 9)	3.01 ± 0.39	0.86 ± 0.12	0.82 ± 0.08	4.03 ± 0.32	1.58 ± 0.14
AZD8835 6mg/kg (n = 9)	3.75 ± 0.44	0.76 ± 0.06	1.29 ± 0.24	3.64 ± 0.49	1.55 ± 0.16
AZD8835 12.5mg/kg (n = 7)	2.50 ± 0.45 *	0.83 ± 0.07	1.07 ± 0.16	4.56 ± 0.71	1.49 ± 0.14
AZD8835 25mg/kg (n = 8)	2.67 ± 0.30 *	1.01 ± 0.12 *	0.66 ± 0.11	3.54 ± 0.39	1.72 ± 0.15
AZD8835 50mg/kg (n = 4)	2.51 ± 0.32 *	1.94 ± 0.34 *	1.97 ± 0.58 *	5.62 ± 0.88	3.14 ± 0.62 *

### A dose response PK/PD relationship for pathway activity is observed

**Fig 3:**
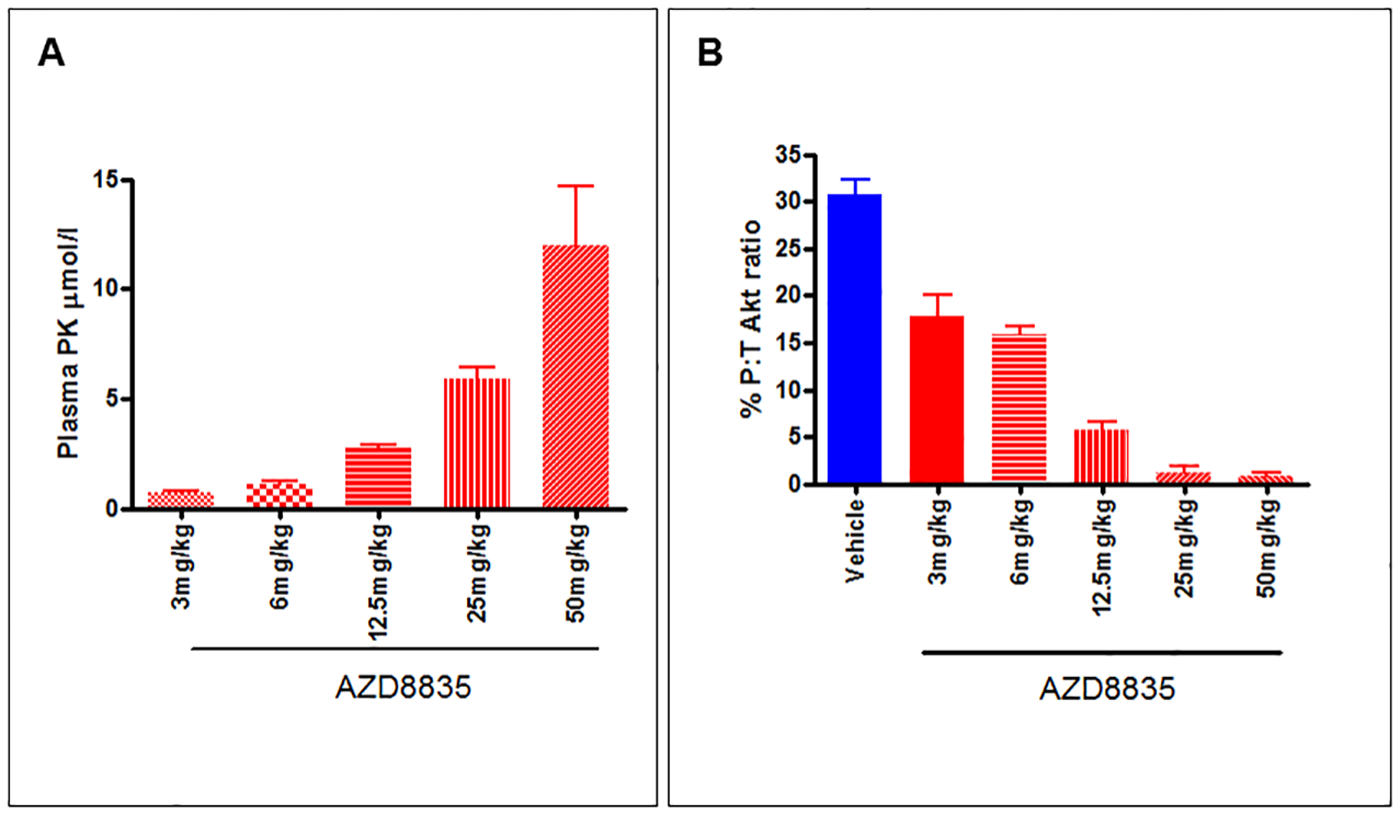
Pharmacokinetic and Meso Scale Discovery Assay (MSD) data demonstrate a good dose response effect following AZD8835 administration. A) Plasma pharmacokinetic analysis following AZD8835 dosing. Vehicle treated animals showed no circulating AZD8835; AZD8835 3mg/kg n = 9; AZD8835 6mg/kg; AZD8835 12.5mg/kg n = 7; AZD8835 25mg/kg n = 8; AZD8835 50mg/kg n = 4. B) MSD assay to assess AKT phosphorylation. Data is expressed as the % of phosphorylated AKT to total AKT. Vehicle n = 8; AZD8835 3mg/kg n = 9; AZD8835 6mg/kg n = 9; AZD8835 12.5mg/kg n = 7; AZD8835 25mg/kg n = 8; AZD8835 50mg/kg n = 4

Pharmacokinetic analysis showed circulating AZD8835 in the blood at every dose level. After 2 hours, levels of circulating AZD8835 increased at each dose with 0.72, 1.13, 2.72, 5.4 and 12.69μmol/l seen at 3, 6, 12.5, 25 and 50mg/kg respectively (Fig 3A) indicating a dose-dependent increase.

MSD based measurement of pAKT, informing of PI3K signalling pathway modulation also showed a dose response effect. Levels of pAKT were decreased at every dose with the ratio of phosphorylated to total AKT being 30.67% in the vehicle treated group and 17.2, 15.8, 5.7, 1.2 and 0.75% in the 3, 6, 12.5, 25 and 50mg/kg treated group respectively (Fig 3B).

### Other pharmacodynamic biomarkers correlate with ^18^F-FDG PET imaging data

Pharmacodynamic activity of AZD8835 was also measured using ELISA and immunohistochemical analysis, providing two measures of phosphorylated PRAS40 signal. A dose response effect was seen using both methods.

**Fig 4:**
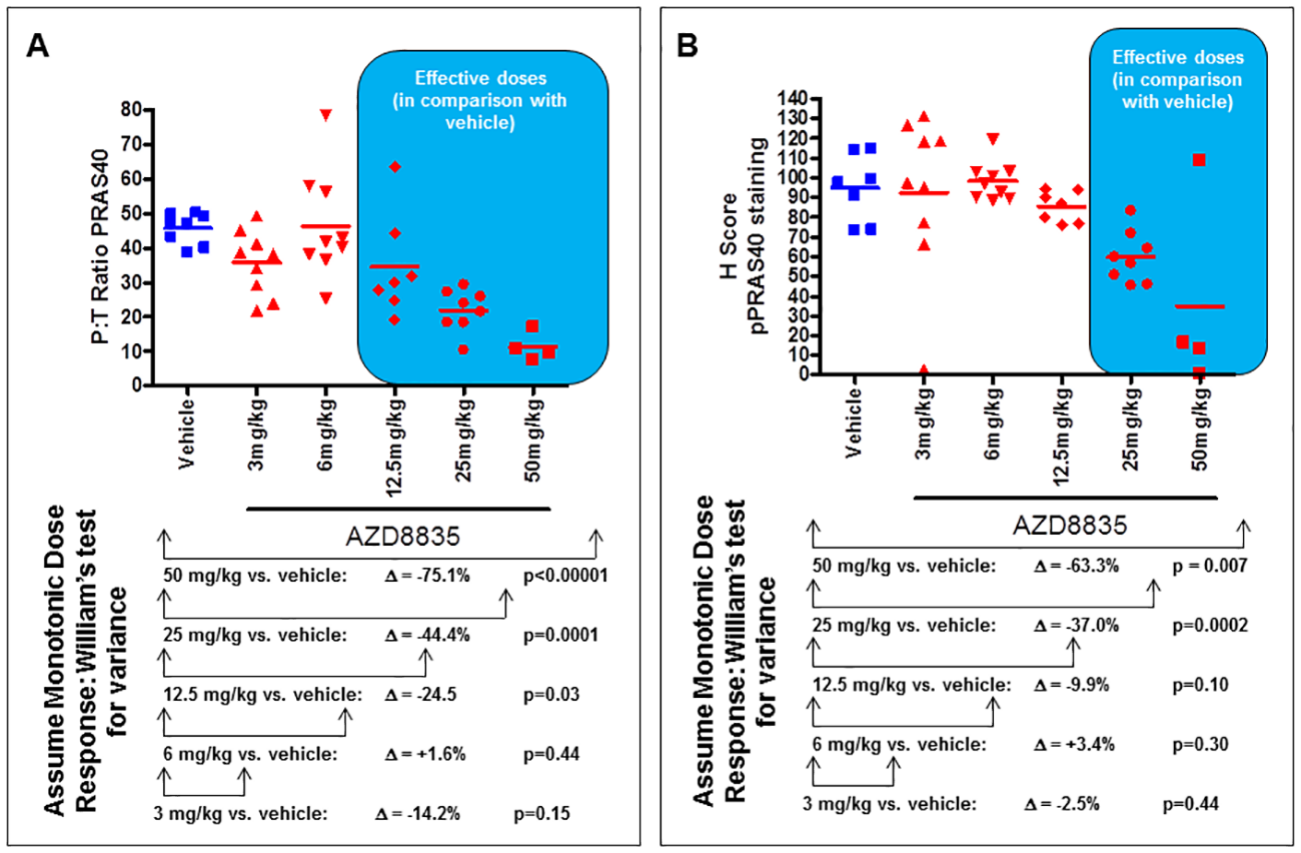
Pharmacodynamic ELISA and immunohistochemistry (IHC) PRAS40 data demonstrate a good dose response effect following AZD8835 administration. A) Plasma pharmacodynamic ELISA analysis following AZD8835 dosing. Data is expressed as the volume of phosphorylated AKT to total AKT expressed as a %. Vehicle n = 8; AZD8835 3mg/kg n = 9; AZD8835 6mg/kg; AZD8835 12.5mg/kg n = 7; AZD8835 25mg/kg n = 8; AZD8835 50mg/kg n = 4. B) IHC pharmacodynamic analysis following AZD8835. Vehicle n = 8; AZD8835 3mg/kg n = 9; AZD8835 6mg/kg n = 9; AZD8835 12.5mg/kg n = 7; AZD8835 25mg/kg n = 8; AZD8835 50mg/kg n = 4

By ELISA, phosphorylated PRAS40 (expressed as a ratio of phosphorylated to total signal) was significantly reduced at 12.5, 25 and 50mg/kg AZD8835 compared to vehicle (24.5%, 44.4% and 75.1% reduction in P:T PRAS40 expression compared to vehicle) (Fig 4A). There was no significant reduction in P:T PRAS40 expression at 3 and 6mg/kg compared to vehicle (1.6% increased and 14.2% decrease in P:T PRAS40 expression respectively compared to vehicle).

The IHC H Score of formalin fixed tumour samples also showed a significant decrease in phosphorylated PRAS40 expression. A significant decrease in the H score was seen at 25 and 50mg/kg AZD8835 compared to vehicle (37.0% and 63.3% decrease in H score compared to vehicle) (Fig 4B). There was no significant decrease in the H score at 3, 6 and 12.5mg/kg AZD8835 compared to vehicle (2.5% decrease, 3.4% increase and 9.9% decrease in the H score of pPRAS40 expression respectively compared to vehicle)

### No effect on glucose homeostasis was seen following AZD8835 administration 2 hours after administration

**Fig 5:**
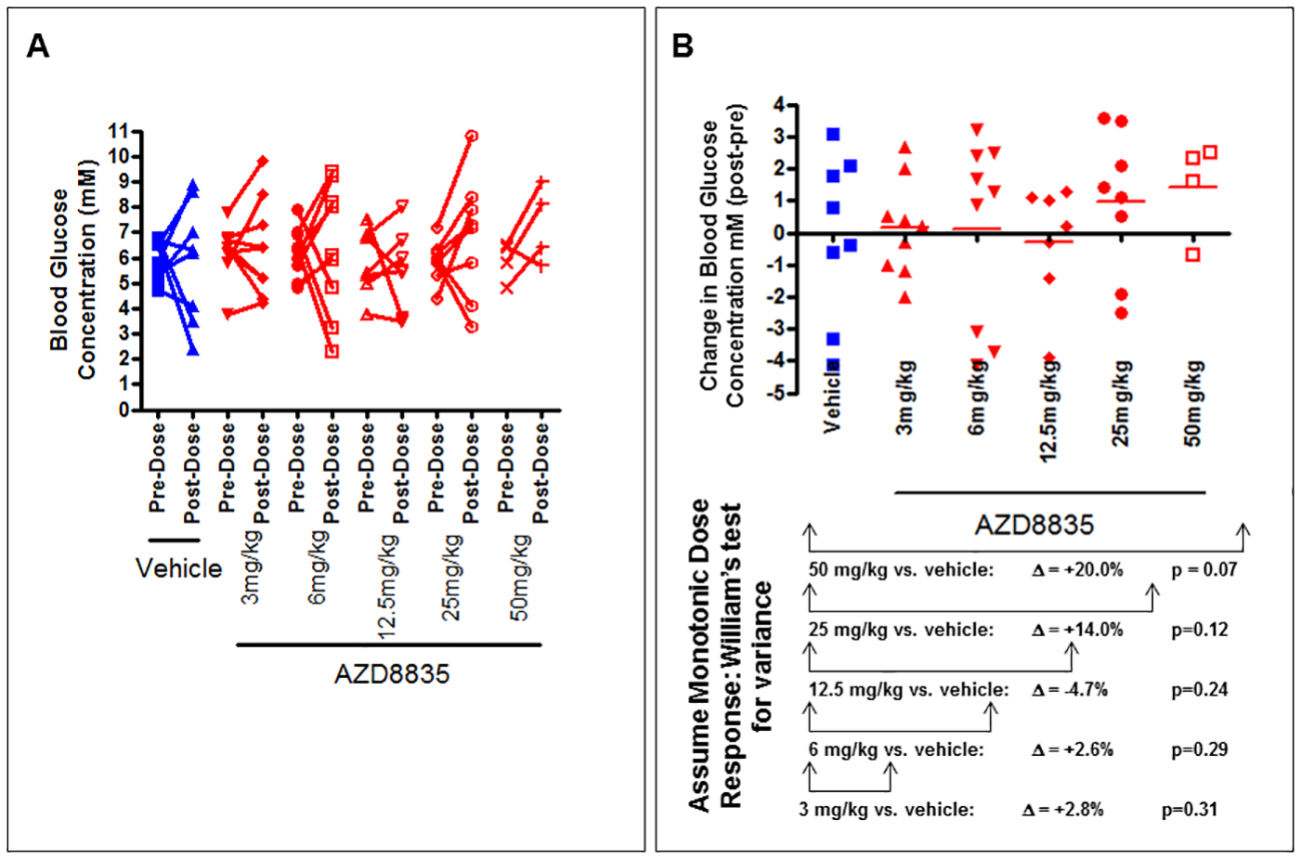
Blood glucose pharmacodynamic data demonstrates a dose response effect following AZD8835 administration with no significant change in glucose homeostasis. A) Blood glucose concentration before and after dosing with AZD8835/vehicle. Data is expressed as the individual animal data. Vehicle n = 8; AZD8835 3mg/kg n = 9; AZD8835 6mg/kg; AZD8835 12.5mg/kg n = 7; AZD8835 25mg/kg n = 8; AZD8835 50mg/kg n = 4. B) The change in blood glucose concentration following AZD8835/ vehicle dosing. Data is expressed as the mean and individual animal data. Vehicle n = 8; AZD8835 3mg/kg n = 9; AZD8835 6mg/kg n = 9; AZD8835 12.5mg/kg n = 7; AZD8835 25mg/kg n = 8; AZD8835 50mg/kg n = 4.

Assessment of blood glucose concentration using a paired t-test showed no significant increase in blood glucose concentration when comparing pre-and post dose measurements in the fasted state (p>0.05 at every dose/vehicle). A trend for an increase in blood glucose concentration was seen at 50mg/kg (p = 0.07 pre-dose vs. post-dose) (Fig 5A). When assessing the change in blood glucose concentration using a Williams test for variance, no significant effect on glucose homeostasis was seen and there was no significant increase in the change in blood glucose concentration at 3, 6, 12.5, 25 and 50mg/kg AZD8835 vs. vehicle (2.8% and 2.6% increase and 4.7% decrease, followed by a 14% and 20% increase compared to vehicle) (Fig 5B). Although the change itself was not significant a trend for a dose response relationship was observed both at 25mg/kg and 50mg/kg (Fig 5B).

## Discussion

We have shown that ^18^F-FDG PET can be used as a pharmacodynamic biomarker with AZD8835. We first used one dose following a single imaging time point of 2 hours to determine if a reduction in ^18^F-FDG uptake existed and furthermore if this correlated with other pharmacodynamic end-points. We found that ^18^F-FDG uptake was reduced by 27% and 23% by 12.5mg/kg dose in imaging and biodistribution data respectively and this correlated well with ELISA and immunohistochemistry data demonstrating inhibition of phosphorylated PRAS40 as a read-out of PI3K pathway activity. We also showed at this pharmacologically active dose of 12.5mg/kg that ^18^F-FDG PET showed tumour specific modulation and no significant changes were seen in any other tissues following AZD8835 administration. This is in contrast with other PI3K inhibitors where an increase in ^18^F-FDG in the blood can also be seen following compound administration [8].

It was hypothesised that ^18^F-FDG PET would be a good biomarker with AZD8835 because of the association of the pathway with glucose metabolism and the use of ^18^F-FDG PET as a PD biomarker with other PI3K pathway inhibitors both clinically [12, 14] and pre-clinically [13, 19]. Specifically it has been shown to be a good biomarker with the PI3K targeted inhibitor GDC-0032 in phase 1 clinical trials. In one study, pharmacodynamic knock-down of PI3K pathway activity was observed at the lowest dose level of 3mg/kg and was shown using both ^18^F-FDG PET and paired tumour biopsies. A partial metabolic response (>25% reduction in ^18^F-FDG uptake compared to baseline) was seen in 18 out of 33 patients (55%) [20]. In a further study using GDC-0032 this time in combination with Fulvestrant, pharmacodynamic knock-down of PI3K pathway activity was observed at the lowest dose level (6mg) and a partial metabolic response was observed in 11 out of 15 (73%) of patients [21].

In order to understand the sensitivity of ^18^F-FDG PET as a pharmacodynamic biomarker with AZD8835 and its potential in phase1 clinical studies we investigated the capability of ^18^F-FDG PET to determine the minimal pharmacologically active dose of AZD8835. *In-vivo* ^18^F-FDG PET imaging revealed significant reductions in ^18^F-FDG uptake at 12.5, 25 and 50mg/kg with no significant reduction in ^18^F-FDG uptake at 3 and 6mg/kg. MaxSUV showed a 34%, 40% and 30% decrease in ^18^F-FDG uptake compared to vehicle and a Max SUV of 1.2, 1.0 and 1.2 in the 12.5, 25 and 50mg/kg treated group respectively. This plateauing of metabolic effect is presumably observed due to the introduction of partial volume effects of the PET scanner. This imaging study also correlated well with the results seen in the first pharmacodynamic study using 12.5mg/kg AZD8835 where a 27% reduction in ^18^F-FDG uptake was seen compared with a 34% reduction in ^18^F-FDG uptake in this dose response study. Biodistribution data in the dose response study also showed a significant reduction in the tumour in the 12.5, 25 and 50mg/kg AZD8835 treated group respectively. At higher doses a significant increase in ^18^F-FDG uptake in the blood was observed at 25 and 50mg/kg and in the muscle and liver at 50mg/kg. This data corresponds nicely with the hypothesis that mechanism of drug induced hyperglycaemia is often observed with PI3K inhibitors, including AZD8835, as a result of altered hepatic glycogen metabolism and blockage of peripheral glucose uptake through GLUT4 [22]. As discussed below in further detail, we also observed elevated glucose at doses of 25 and 50mg/kg AZD8835, albeit not statistically significant.

In order to further validate the ^18^F-FDG PET imaging data, we analysed and investigated the pharmacokinetic effect of AZD8835 in a dose dependent manner and its pharmacodynamic effects with other clinically relevant pharmacodynamic biomarkers.

Pharmacokinetic analysis showed a good dose response effect following AZD8835 administration. A Meso Scale Discovery assay measuring AKT phosphorylation (S473) and informing of AZD8835 activity also demonstrated a good dose response effect with AZD8835.

Phosphorylation of PRAS40, a further downstream marker of PI3K pathway activity, and a good clinically available PD biomarker [23] was also measured with ELISA and immunohistochemistry analysis. Using ELISA analysis a significant decrease in PRAS40 phosphorylation (T246) was observed for tumours treated with AZD8835 at 12.5, 25 and 50mg/kg. Using immunohistochemistry analysis a significant difference in PRAS40 phosphorylation was observed for tumours treated with AZD8835 at 25 and 50mg/kg. Therefore ^18^F-FDG PET and ELISA analysis appear to be more sensitive than immunohistochemistry at detecting pPRAS40 based measurement of PI3K pathway activity with ^18^F-FDG PET having the advantage of providing this sensitivity via a non-invasive approach.

A further potential pharmacodynamic biomarker for AZD8835 is the use of blood glucose as a systemic biomarker of PI3K activation [8]. Although a mechanism based toxic effect associated with PI3K/AKT/mTOR inhibitors, hyperglycaemia is often used as a pharmacodynamic biomarker for clinical decision making and dose setting. Because PI3K is a key component of the insulin signalling pathway, then inhibition of insulin signalling leads to insulin resistance and thus increased glucose levels in the blood [24]. In this study we showed a trend towards but not quite a statistically significant change in blood glucose concentration after any dose of AZD8835. This contrasts with mouse studies elsewhere reporting glucose elevation by 25mg/kg AZD8835 [8]. However there are several differences between these studies. Firstly, in this current study, imaging and glucose measurement were 2 hours after dosing, but previous data [8] highlights the transient nature of glucose elevations and maximal signal at 0.5 rather than 2hrs. Secondly, different mouse strains were used in these AZD8835 studies. Thirdly, in this current study animals were fasted which may attenuate the drug induced hyperglycaemia with PI3K inhibitor [22]. Finally, only 4 animals received the 50mg/kg treated group in this study, thereby weakening the statistical significance value.

The fact that we see no significant change in blood glucose in this study however gives us re-assurance however that there are no competing effects on ^18^F-FDG uptake in the tumour from elevated systemic glucose.

These observations of a dose dependant change in ^18^F-FDG uptake are consistent with pre-clinical reports elsewhere where ^18^F-FDG PET was used to determine the biological dose of the mTOR inhibitor Everolimus [25]. In this study, they sought to test whether quantitative changes in tumour ^18^F-FDG uptake would qualify as a surrogate endpoint for predicting the anti-tumour activity of Everolimus and concluded that metabolic changes elicited by Everolimus and visualised by PET are closely linked to the anti-tumour activity, where dose escalation beyond 5mg/kg/day did not result in an enhanced anti-tumour effect. In the study, the validity of ^18^F-FDG uptake as a surrogate marker was supported by other PD marker data where cancer cell proliferation measured by Ki67 expression also favoured the same optimum biological dose of the ^18^F-FDG PET data. Consistent with the study by Cejka *et al*, we too show that the ^18^F-FDG PET changes seen at 12.5, 25 and 50mg/kg are linked closely to the anti-tumour activity of AZD8835 shown in the efficacy growth curve in the paper by Barlaam *et al* [7].

At doses lower than 25mg/kg AZD8835 a significant increase in blood glucose following AZD8835 administration would not be expected and therefore it is hypothesised that ^18^F-FDG PET could be a more sensitive biomarker than blood glucose alone. This correlates with other studies where the use of plasma glucose as a pharmacodynamic biomarker with PI3K inhibitors has also shown to be less sensitive than other markers such as ^18^F-FDG PET and tumour biopsy. In the GDC-0032 study using GDC-0032 alone [20] hyperglycaemia was only observed in patients at 8mg/kg but a PD read-out with ^18^F-FDG PET and paired biopsy was seen at 3mg/kg, similarly in the combination study using GDC-0032 and Fulvestrant [21] at the lowest dose level where significant PD response was observed with ^18^F-FDG PET and paired biopsy hyperglycaemia was only present in 29% of patients.

The use therefore of ^18^F-FDG PET may be a good option in clinical studies to assess target engagement in tumour tissue in the clinic. It can be used to complement IHC based dynamic biomarker readouts where if paired tumour biopsies are available, or as an alternative if it proves difficult to obtain biopsies.

These studies amongst others highlight the potential of ^18^F-FDG PET as a clinical biomarker. In particular, they illustrate the use of pre-clinical studies to evaluate the suitability of ^18^F-FDG PET as a clinical biomarker, including measures of sensitivity compared with alternative endpoints. It could also serve as an important biomarker in the pre-clinical setting, through contribution to pre-clinical effective dose setting.

In conclusion, our pre-clinical studies support the use of ^18^F-FDG PET imaging as a sensitive and non-invasive pharmacodynamic biomarker (understanding the role of PI3K signalling in glucose uptake) for AZD8835 with a decrease in ^18^F-FDG uptake observed at only two hours post treatment. The decrease in ^18^F-FDG uptake was dose dependent and data showed excellent PK/PD correlation. This data supports and parallels observations obtained with this class of compounds in patients [20,21].

## Supporting information

S1 Fig All raw data corresponds to the manuscript data. (XLSX)
